# Comparison of the optical behaviour of five different multifocal diffractive intraocular lenses in a model eye

**DOI:** 10.1038/s41598-023-47102-y

**Published:** 2023-11-10

**Authors:** Efe Can, Esat Can Senel, Sven T. S. Holmström, David P. Piñero

**Affiliations:** 1 R & D Department, VSY Biotechnology GMBH, Esslinger Str. 7, 70771 Leinfelden Echterdingen, Germany; 2 Group of Optics and Visual Perception, Department of Optics, Pharmacology and Anatomy, University of Alicante, Crta San Vicente del Raspeig S/N, 03690 San Vicente del Raspeig, Alicante, Spain; 3 Department of Ophthalmology, Vithas Medimar International Hospital, Alicante, Spain

**Keywords:** Optics and photonics, Preclinical research

## Abstract

The purpose of this study was to investigate and compare the optical performance of five trifocal intraocular lenses (IOLs) following the ISO 11979-2 standards, analysing the impact of tilt and decentration. Five different diffractive trifocal IOLs were evaluated in this experimental study: Acriva Trinova (VSY-Biotechnology) (AT), FineVision HP (PhysIOL) (FVHP), AT LISA tri 839 MP (Zeiss) (ATLT), PanOptix TFNT00 IOL (Alcon) (PO), and Tecnis Synergy (J&J Vision) (TS). In-vitro optical quality analysis of them was performed with the Lambda PMTF system that has an aberration neutral cornea model (Lambda-X Ophthalmics). Measurements were performed on-axis, with 5º of IOL tilt and with 0.5 mm of IOL decentration using 543-nm monochromatic light. Finally, IOL dimensions and diffractive disk profile inspection was performed using the VisIOLA system (Rotlex). On-axis measurements showed a far through-focus MTF > 0.3 at 3 mm aperture, except for TS. FVHP and PO showed better far MTFs for larger apertures (3.75 mm and 4.5 mm) while AT showed good intermediate and near vision for such apertures. With 5º of IOL tilt, the better optical performance at all distances was found with AT for medium-sized pupils (3 mm) and an important reduction of MTF was found for ATLT and PO, especially in the intermediate focus. The induction of 0.5 mm of IOL decentration especially affected the intermediate focus of ATLT and TS and the far focus of FVHP and PO. IOL dimensions and diffractive profile were consistent with those described by the manufacturer. In conclusion, there are differences in the optical performance according to the pupil aperture of the five trifocal IOLs evaluated and this should be considered in clinical practice when selecting the most appropriate implant in each specific case. IOL tilt and decentration can affect significantly in most of the designs evaluated the performance of the IOL at intermediate vision range. It should be noted that measurements were made with an aberration-free cornea, being necessary future studies analysing the impact of different levels of corneal aberrations.

## Introduction

Crystalline lens extraction with implantation of a multifocal intraocular lens (IOL) has been shown to be a safe and cost effective way to provide a complete visual restoration in patients undergoing cataract surgery^1^ or clear lens extraction surgery for presbyopia correction.^2^ Such lenses provide several foci allowing a functional vision at different distances that differ according to their optical design.^3^ Among them, diffractive trifocal IOLs are one of the most commonly used implants in the clinical practice. The trifocal IOLs tested in this study were all hybrid refractive-diffractive lenses, using a diffractive surface in one side of the lens to distribute light to several diffractive orders. It is often assumed that a lens always produces three dominating diffractive orders directly corresponding to the three main focal distances of clinical interest, far, intermediate and near, but this is not always the case.^3^ Depending on the design of the diffractive surface, the IOL will have a specific optical behaviour to provide the patient with a differential visual performance.^4–10^ For this reason, optical bench comparative studies are performed prior to clinical studies to investigate and analyse the differences in the optical performance between different trifocal implants, allowing clinicians to infer the potential differences in visual performance.^1, 7, 8^

Some studies indicate that trifocal IOLs are more sensitive to tilt and decentration.^11–13^ However, there is no consensus in the literature, with at least one study suggesting that the opposite trend could be possible.^5^ Likewise, most of trifocal IOLs introduce asphericity in one of the surfaces of the lens in order to induce some level of negative spherical aberration that might enhance the image quality by neutralizing the positive spherical aberration normally caused by the cornea. However, this can make the IOLs less tolerant to tilts or decentrations.^12, 13^ Some levels of IOL tilt (2º–3º) and decentration (0.2–0.3 mm) are commonly found in clinical studies that are unnoticed by most of patients, even in patients implanted with multifocal designs.^14^ Baumeister et al^15^ found with Scheimpflug photography of implanted aspheric IOLs a mean optic tilt of 2.85 degrees ± 1.36 (SD) and a mean decentration of 0.27 ± 0.16 mm. It is important for IOLs to function within the commonly occurring range of tilt and decentration.^15^ According to simulations and considering aspects such as corneal aberrations, pupil function, and other factors influencing the visual performance, IOL decentrations of 0.5 mm can lead to significant visual degradation.^16^ Tandogan et al^11^ found in another experimental study that optical quality was significantly reduced at all distances for diffractive bifocal and trifocal IOLs if the IOL decentration exceeded 0.75 mm, with intermediate focus showing the least reduction.

MTF measurements at multiple apertures could be a very important way for understanding lens performance in different lighting conditions and with regards to the activities often carried out in these specific conditions.^17^ Pupil size is dependent on at least age, lighting condition, and focus distance. Outdoor activities in strong sunlight are related to very small pupil sizes, especially for the oldest patients, as well as reading in well-lit environments, whereas night driving will be performed with a higher level of mydriasis. Trifocal lenses should be designed considering the interaction between IOL light distribution and pupillary changes occurring at different ages and under different conditions. Therefore, it is very important to measure the pupil dependency of multifocal IOLs.

The current experimental study was carried out to investigate the optical performance of five trifocal IOLs following the ISO 11979-2 standards, analysing for different pupil sizes the impact of IOL tilt and decentration with all of them.

## Methods

### Intraocular lenses

A total of 5 different diffractive trifocal IOLs were evaluated in this study at the Laboratory of R&D Department of VSY Biotechnology (Table 1). For FineVision HP, AT LISA tri 839 MP, PanOptix TFNT00 and Tecnis Synergy designs, the IOL tested had an optical power of 21 D, whereas an IOL of 20 D was tested for Acriva Trinova Pro C.

**Table 1:** Main characteristics of the five diffractive trifocal intraocular lenses evaluated in the current experimental study.

Trade Name (Company)	Type	Add (D)	SA (µm)	Refractive index	Abbe number
Acriva Trinova Pro C (VSY Biotechnology)	Hydrophilic	+ 1.8/ + 3.6	− 0.10	1.46	58
FineVision HP (PhysIOL)	Hydrophobic	+ 1.75/ + 3.50	− 0.11	1.52	42
AT.LISA tri 839 M (Carl Zeiss Meditec)	Hydrophilic	+ 1.66/ + 3.33	− 0.18	1.46	56.5
AcrySof PanOptix TFNT00 (Alcon)	Hydrophobic	+ 2.20/ + 3.20	− 0.10	1.55	37
TECNIS Synergy Optiblue (Johnson & Johnson VISION)	Hydrophobic	Enhanced depth of field	− 0.27*	1.47	55

*The value for spherical aberration of the Tecnis Synergy is not explicitly given, but is assumed to be close to -0.27 µm since it is stated to be designed to “reduce corneal spherical aberration to near-zero”.

The Acriva Trinova IOL (VSY Biotechnology, Germany) is a one-piece trifocal IOL. The diffractive portion of the IOL has a sinusoidal design and is located on its anterior surface, whereas the posterior surface is purely refractive (optic size 6.0 mm). With the sinusoidal design, diffraction can be achieved with fewer diffractive rings and without the sharp corners, allowing tuning the light distribution at each aperture by changes to the continuous diffractive profile. It provides an addition of + 1.8 D for the intermediate focus and + 3.6 D for the near focus (at the IOL plane). For pupils below 1.4 mm, the light is almost fully distributed to far vision. This zone is motivated by the Stiles-Crawford effect of the first kind (“directional sensitivity of the retina”): axial rays entering the pupil near center are more effective than off-axis rays. For increasing pupil sizes, the energy to the near vision increases reaching a light distribution of 41% for far, 24% for intermediate, and 35% for near for a 3 mm aperture, and a far/intermediate/near light distribution of 43%, 34%, and 24% for a 4.5 mm aperture.^18^.

The FineVision HP IOL (PhysIOL, Liège, Belgium) is a one-piece, trifocal, aspheric diffractive IOL. It is based on the bi-bi principle, which essentially refers to the combination of a near add (3.50 D) bifocal diffractive profile with an intermediate add (1.75 D) apodized lens. The FineVision lens makes use of an overlapping and strongly apodized sawtooth diffractive pattern.^19^ Apodization means that the height of the diffractive profile decreases with increasing radius and is an efficient way in sawtooth lenses to move light intensity to the far focal point from the other foci. The IOL has a total size of 11.40 mm and an optic diameter of 6.0 mm. It is made of a glistening-free material (PhysIOL G-free) and has an improved haptics design that allows easier separation in the eye.^20^ According to our measurements, the light energy distribution is 48%, 19% and 33% for far, intermediate, and near, respectively, for a 3 mm aperture. However, for a 4.5 mm aperture, the far energy distribution increases up to 64%, while the intermediate and near energy drop to 13% and 23%, respectively.

The AT LISA tri 839 MP (Carl Zeiss Meditec, Jena, Germany) is a one-piece diffractive trifocal IOL that has an overall length of 11.0 mm and a 6.0 mm biconvex optic. It is trifocal within an IOL diameter of 4.3 mm, whereas it is bifocal between 4.3 mm and 6 mm (+ 1.66 D addition)^21^. The diffraction pattern makes use of a proprietary technology that Zeiss calls Smooth Micro Phase, rendering smoother corners than in conventional sawtooth patterns. It has been suggested that smoother diffractive profiles can decrease undesired photic phenomena due to less light scattering and could be more biocompatible compared to sawtooth profiles because of reduction in the debris precipitation effect.^22^ According to our measures, the light energy distribution is 41%/27%/33% (far/intermediate/near) and 37%/28%/35% for 3 and 4-mm apertures.

The PanOptix TFNT00 IOL (Alcon, Fort Worth, TX, USA) is a single-piece ultraviolet and blue light filtering, non-apodized, foldable presbyopia-correcting IOL, with a central optic of 6.0 mm and a total diameter of 13.0 mm. The posterior surface of the IOL is spherical, and the anterior surface is aspheric with a diffractive surface on the central 4.5 -mm portion of the optic zone. This diffractive part uses a proprietary optical technology (Enlighten) to, in their terms, “redistribute” the focal point at 120 cm (0.83 D) to the distance focal point for amplified performance, with two additional foci: intermediate at 60 cm (1.67 D) and near at 40 cm (2.5 D). The sawtooth profile used by Enlighten is a bit different from the more known overstepping type of FineVision and renders the lens quadrifocal, as FineVision design uses the 0th order for far vision, but the 1st diffraction order is suppressed, with a very low diffraction efficiency.^22^ The term redistribution used by Alcon refers to the suppression of the 1st order intensity. This creates a different power distribution of the intermediate and near additions. Despite the near addition power being double that of the intermediate addition, which has historically been the case in trifocal lenses, the near and intermediate additions have a 3:2-relationship, enabling the intermediate vision to be as close as 60 cm. For an aperture of 4.5 mm, the far/intermediate/near light energy distribution with this IOL is 58%/19%/22%. For an aperture of 3 mm, the energy distribution is 58%/19%/22% (far, intermediate, near). For larger apertures (3.75 mm and 4.5 mm), there is a significant pupil dependency to favour far vision for scotopic conditions.^9^ In the current study, the usable energy is being considered when energy distribution is discussed, and consequently the sum of the energy distribution to the available foci is 100%.

Finally, the Tecnis Synergy IOL (Johnson & Johnson Vision, Santa Ana, CA, USA) is a one-piece presbyopia-correcting IOL with a 6-mm optic and an overall length of 13 mm. It uses a proprietary diffractive design that results from the combination of the diffractive technologies of Tecnis multifocal (bifocal diffractive profile) and Tecnis Symfony IOLs (diffractive-based EDOF design),^23^ with an unusually high step height close to the centre of the lens.^24^ Additionally, this is the only multifocal lens of the ones measured here that is designed to correct for the full spherical aberration of the human eye, which is considered as + 0.27 µm^25^. The Synergy lens should be expected to perform comparatively better in systems where positive spherical aberration is introduced. According to our measures, the energy distribution for a 3 mm aperture is 43%/20%/37% (far/intermediate/near) and 43%/21%/36% (far/intermediate/near) for a 4.5 mm aperture.

### Experimental setup and metrics

The Lambda PMTF device (Lambda-X Ophthalmics, Nivelles, Belgium) was used to assess the in-vitro image quality of the five multifocal diffractive IOLs evaluated. This device is capable of taking optical measurements in compliance with ISO 11979-2.^26^ Briefly, light of wavelength of 543 nm follows the following path within the optical system: resolution target, collimating lens, aperture, ISO eye model 1 (aberration-free eye model), IOL immersed in a wet cell with balanced salt solution, X20 microscope (spatial effective resolution of 0.23 μm/pixel), and CCD camera (pixel size of 4.65 × 4.65 μm). This device utilizes the slanted edge technique to calculate the modulation transfer function (MTF), which consists in imaging an edge onto the detector.^27^ It should be considered that the IOL placed inside an 11.0 mm diameter holder that allows the control of misalignments and tilts. Horizontal edge image was utilized in the tests.

Besides the aberration-less eye model (ISO eye model 1), ISO 11,979–2 also describes Eye Model 2, which allows for modelling an aberrated cornea. Most commonly used is a cornea with a spherical aberration of 0.27 µm, since this is understood as the human average. There is no consensus in the literature on which is the single most suitable mode to compare lens performance. While it is clear that the average human eye has higher spherical aberration than the neutral model, lenses optimised for the fully aberrated model become more sensitive to decantation and tilt and it has been argued that lenses that perform better in the neutral model will perform well for a larger range of different eyes.^28^ It has been shown that using only a perfectly well-placed lens with a fully aberrated cornea model are misleading.^13^ Because of this, it is the understanding of these authors that Eye Model 1 remains at the very least one good way to compare lenses, even it has to be kept in mind that it does not accurately demonstrate the qualities of fully aberration correcting lenses.

In the current experimental study, the measurements with the Lambda PMTF device were done following the same specific sequence for each IOL. First, the IOL was placed into a crystal cuvette containing saline solution at room temperature in a position where its front surface was facing downwards. Then, using the XY translational stage, the lens was centered and an on-axis measurement was obtained. After this, tilted and decentered measurements were done after adjusting the corresponding knobs. Specifically, measurements were obtained for an IOL tilt of 5°, and also inducing an IOL decentration of 0.5 mm. For each orientation, MTF graphs at 50 Lp/mm and USAF (United States Air Force) resolution images were acquired at 2 mm, 3 mm, 3.75 mm, and 4.5 mm apertures. The through focus MTF (TF-MTF) measurement was obtained, which provides the MTF at a contiguous range of focus planes. It should be considered that it is of special interest for multifocal lenses the analysis of the IOL performance at distances corresponding to far, intermediate, and near vision, as these positions are of clinical interest.^29^ Clinically, it is common to test near vision at a distance of 40 cm and the intermediate vision at a distance of 66 cm. However, near vision is generally considered as the range between 20 and 40 cm and intermediate vision as the range between 50 and 80 cm.^29^ In terms of optical power, the above given range for near corresponds to an additional power (over emmetropic far vision) from 3.6 to 7.0 D at the lens plane, corresponding to 4.7–2.5 D at the corneal plane. The intermediate range is defined from 2.9 to 1.8 D, corresponding to a range from 2.0 to 1.2 D at the corneal plane. However, some trifocal lenses have additions that are slightly weaker than those contained in these ranges. In the current study, the power of lens addition is always referred to the lens plane if nothing else is specified.

Concerning the measurement of energy distribution, it was estimated considering that the Strehl ratio (SR) is the usable energy in the focused spot and that this SR can also be calculated from the MTF curves as the quotient of the area under actual MTF curve to the area under diffraction limited MTF curve. The Lambda PMTF device measures one MTF curve at each focus and defines the energy in each focus as the SR on this focus divided by the sum of SR in all foci.

All these optical quality measures (NTF, SR and energy distribution) were based on a single measurement. One lens was measured for each model. Though, before taking the final measurements, the IOLs were measured multiple times to ensure that measurement stability.

Besides the analysis of the optical performance of each IOL, an additional analysis of IOL dimensions and diffractive disks was performed using the VisIOLA system (Rotlex, Omer, Israel). This device combines fine optics and specialised software to create a detailed inspection of any IOL, allowing the technician to measure the diameter of the optics, the overall size, the distance between the positioning holes and their diameters, and the haptic width.

## Results

### General data

**Figure 1:**
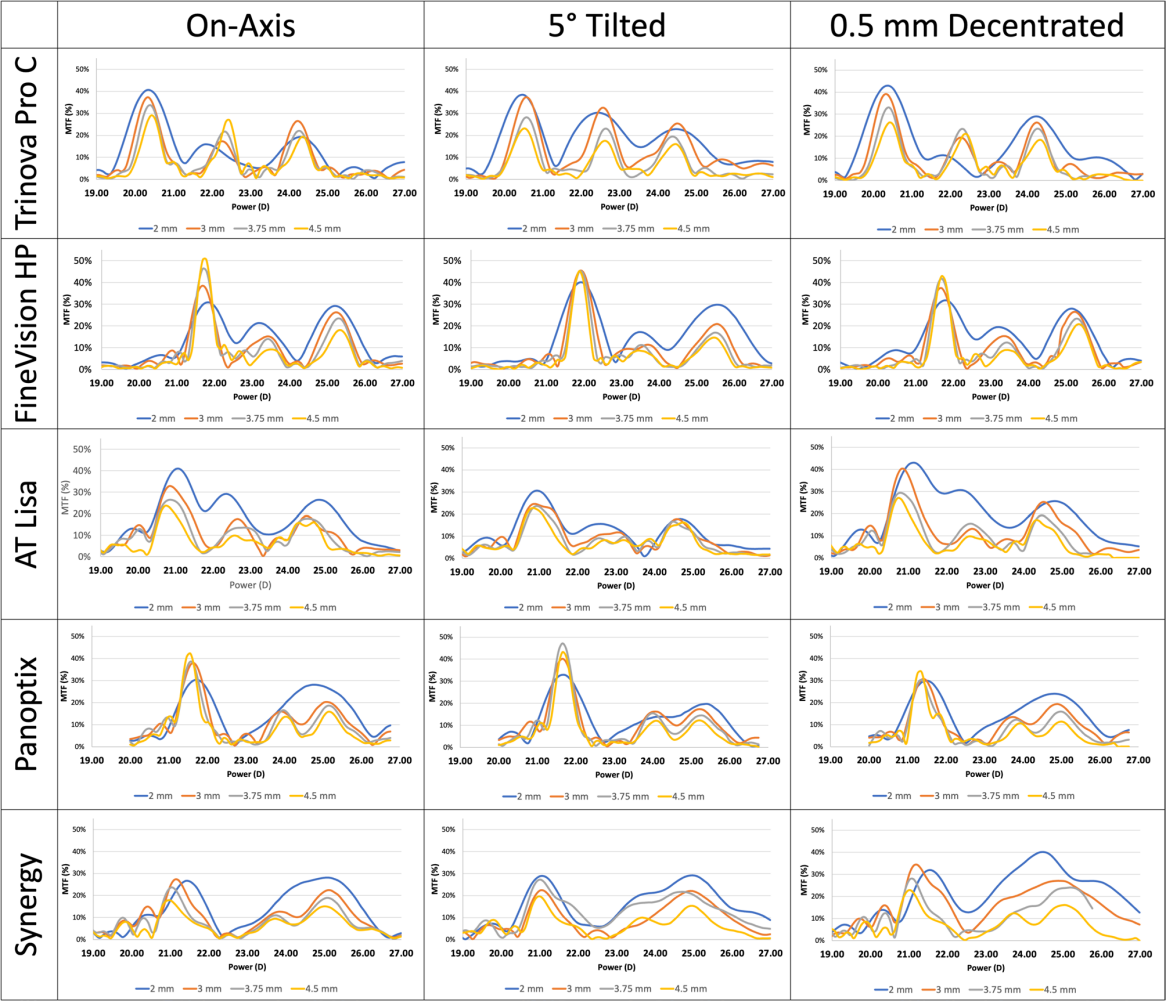
Experimental modulation transfer function (MTF) graphs obtained with each intraocular lens (IOL) evaluated for the spatial frequency of 50 Lp/mm and for the pupil apertures of 2 mm, 3 mm, 3.75 mm, and 4.5 mm. All IOLs had an optical power of 21 D.

Figure 1 shows the MTF graphs at 50 Lp/mm acquired at 2 mm, 3 mm, 3.75 mm, and 4.5 mm apertures for each IOL tested (all of them of 21 D). All this information in combination with energy distribution is described in detail in Table 2.

**Table 2:** Optical performance data obtained in the experimental measures on-axis with the Lambda PMTF device for the five intraocular lenses (IOLs) evaluated.

Trade Name (Company)	Aperture (mm)	Energy distribution			MTF			Lens power (D)		
		*Far*	*Int*	*Near*	*Far*	*Int*	*Near*	*Power*	*Int Add*	*Near Add*
Acriva Trinova Pro C (VSY Biotechnology)	*2* *3* *3.75* *4.5*	0.43 0.41 0.39 0.42	0.23 0.24 0.29 0.34	0.34 0.35 0.20 0.24	0.41 0.37 0.33 0.29	0.16 0.17 0.22 0.27	0.19 0.26 0.26 0.22	20.25 20.21 20.23 20.20	1.10 1.86 1.63 1.31	3.75 3.66 3.64 3.66
FineVision HP (PhysIOL)	*2* *3* *3.75* *4.5*	0.37 0.48 0.56 0.64	0.26 0.19 0.16 0.13	0.36 0.33 0.28 0.23	0.31 0.39 0.46 0.50	0.21 0.15 0.14 0.10	0.29 0.26 0.23 0.18	21.6 21.43 21.32 21.25	1.42 1.75 1.79 1.53	3.25 3.45 3.51 3.59
AT.LISA tri 839 M (Carl Zeiss Meditec)	*2* *3* *3.75* *4.5*	0.41 0.41 0.44 0.37	0.30 0.27 0.23 0.28	0.29 0.33 0.33 0.35	0.41 0.33 0.26 0.24	0.29 0.18 0.14 0.16	0.26 0.19 0.18 0.16	21.00 20.69 20.71 20.72	1.08 1.71 1.91 2.57	3.49 3.40 3.25 3.58
AcrySof PanOptix TFNT00 (Alcon)	*2* *3* *3.75* *4.5*	0.52 0.48 0.50 0.58	– 0.21 0.24 0.19	0.48 0.31 0.26 0.22	0.30 0.38 0.39 0.42	– 0.16 0.17 0.14	0.28 0.20 0.19 0.16	21.14 20.98 20.84 21.56	– 1.93 2.17 2.50	2.82 3.10 3.12 3.83
TECNIS Synergy Optiblue (Johnson & Johnson VISION)	*2* *3* *3.75* *4.5*	0.45 0.43 0.44 0.43	0.00 0.20 0.20 0.21	0.55 0.37 0.35 0.36	0.29 0.28 0.24 0.18	– 0.13 0.11 0.09	0.36 0.24 0.19 0.15	19.97 19.98 20.73 20.75	1.19 1.83 2.49 2.52	4.61 4.41 3.71 3.71

*MTF* modulation transfer function, *Int* intermediate, *Add* addition.

### On-axis optical performance data

As shown in Fig. 1, a trend to better on-axis MTF at far was observed for smaller pupils (2 mm and 3 mm diameters) with the trifocal IOLs Acriva Trinova, AT LISA tri 839 MP and Tecnis Synergy. In addition, better intermediate and near on-axis MTFs was observed with the smallest aperture for all the IOLs, except the Acriva Trinova for which the near vision performance increased slightly up to a 3-mm aperture, and the intermediate performance increased with increasing aperture. 

The far energy levels with the Acriva Trinova IOL at different apertures was almost maintained, with a dropping trend in the MTF at near and far foci (Fig. 1, Table 1). Compared to the other four IOLs, the Acriva Trinova IOL showed the largest intermediate MTF value for larger apertures (3.75 mm and 4.5 mm). In contrast, the FineVision HP IOL showed a far/intermediate/near light distribution of 48%/19%/33% for a 3 mm aperture, with a steady increase in energy and MTF at far focus for larger apertures. However, with this IOL, better near and intermediate MTFs were found for the smaller apertures. For 2 mm aperture, the far and near foci were very close to each other (MTF of 0.31 and 0.29, respectively) (Fig. 1, Table 2).

The AT LISA tri IOL showed better optical quality for far, intermediate and near foci at the smallest aperture (2 mm). Specifically, this IOL achieved the better MTF for the far and intermediate foci at a 2 mm aperture. For other apertures, the MTF value corresponding to the near focus slightly decreased, but remaining within optimal levels. However, for the intermediate and far foci, the MTF dropped steadily with increasing apertures (Fig. 1, Table 2). Regarding the PanOptix IOL, the measurements conducted with the PMTF device showed that the lens acted as a bifocal lens for a 2 mm aperture, with an energy distribution of 0.52/0.48 (far/near) and MTF of 0.3/0.28 (far/near). An important distinction observed with the PanOptix IOL is that the peaks for intermediate and near vision merged together. For an aperture of 3 mm, the far/intermediate/near energy distribution was 0.48/0.21/0.31, and the far/intermediate/near MTF was 0.38/0.16/0.20. With increasing apertures, far vision MTF increased whereas the near vision decreased, with almost constant values for intermediate focus (Table 2). Finally, the Tecnis Synergy IOL showed for a 2 mm pupil aperture two differentiated peaks, one for far and another one combining the intermediate and near foci, similar to the behaviour of the PanOptix, but even more pronounced. For larger apertures, which are 3.75 mm and 4.5 mm, the near and intermediate MTF peaks became separated, being the near MTF higher than the intermediate. Overall, the MTFs gradually decreased for larger apertures and the intermediate MTF for larger apertures was observed to be very low (Fig. 1, Table 2).

**Figure 2:**
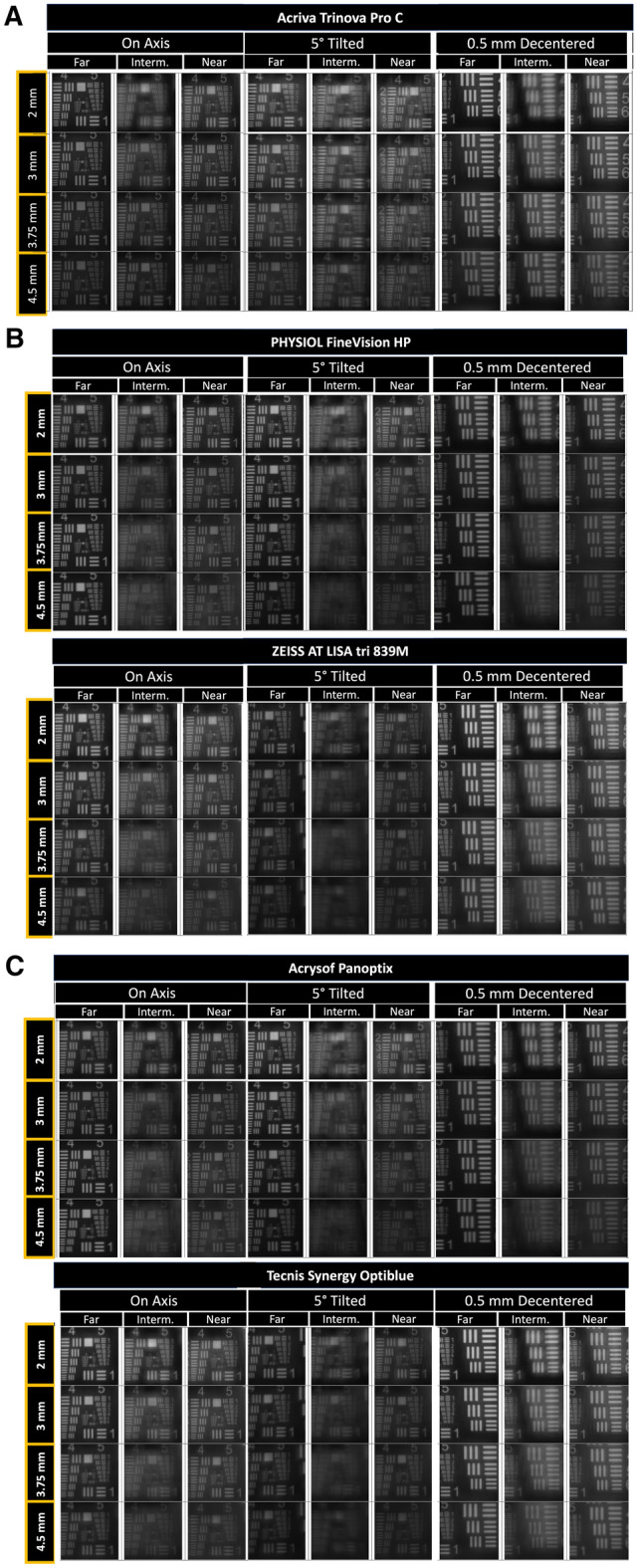
USAF (United States Air Force) resolution images obtained with each intraocular lens (IOL) evaluated for the spatial frequency of 50 Lp/mm and for the pupil apertures of 2 mm, 3 mm, 3.75 mm, and 4.5 mm. All IOLs had an optical power of 21 D. **a** Displays the images pertaining to AT, **b** the FVHP and ATLT, while **c** displays the images for PO and TS.

All these observations were also confirmed when using the USAF (United States Air Force) resolution images to simulate the image generated by the 5 IOLs evaluated, as shown in Figs. 2a, b and c.

Concerning the impact of IOL tilt on optical performance, different trends were observed between IOLs (Fig. 1). For example, the better optical performance at all distances with the Acriva Trinova was found for medium-sized pupils (3 mm) and an important reduction of MTF was found for AT LISA and PanOptix IOLs, especially in the intermediate focus. The tilt of 5º generated light loss at the intermediate and near foci for the FineVision HP and PanOptix IOLs (especially for larger apertures) (Table 3).

**Table 3:** Optical performance data obtained in the experimental measures simulating a tilt of 5º with the Lambda PMTF device for the five intraocular lenses (IOLs) evaluated.

Trade Name (Company)	Aperture (mm)	Aperture	Energy distribution			MTF			Lens power (D)		
			*Far*	*Int*	*Near*	*Far*	*Int*	*Near*	*Power*	*Int Add*	*Near Add*
Acriva Trinova Pro C (VSY Biotechnology)	*2* *3* *3.75* *4.5*	2 mm 3 mm 3.75 mm 4.5 mm	0.37 0.34 0.32 0.37	0.30 0.34 0.34 0.35	0.33 0.25 0.34 0.28	0.38 0.37 0.28 0.23	0.30 0.32 0.23 0.17	0.23 0.25 0.19 0.16	20.31 20.44 20.28 20.32	1.94 1.62 1.73 1.85	3.81 3.45 3.62 3.38
FineVision HP (PhysIOL)	*2* *3* *3.75* *4.5*	2 mm 3 mm 3.75 mm 4.5 mm	0.44 0.60 0.64 0.66	0.23 0.15 0.16 0.16	0.33 0.25 0.17 0.19	0.40 0.45 0.45 0.45	0.17 0.11 0.11 0.09	0.30 0.20 0.17 0.15	21.56 21.50 21.49 21.43	1.73 2.22 1.64 1.47	3.66 3.75 3.46 3.49
AT.LISA tri 839 M (Carl Zeiss Meditec)	*2* *3* *3.75* *4.5*	2 mm 3 mm 3.75 mm 4.5 mm	0.41 0.44 0.47 0.39	0.28 0.24 0.19 0.25	0.31 0.32 0.34 0.35	0.31 0.25 0.24 0.23	0.16 0.12 0.10 0.09	0.18 0.18 0.17 0.16	20.61 20.61 20.76 20.31	1.95 1.98 2.06 2.09	3.62 3.47 3.20 3.85
AcrySof PanOptix TFNT00 (Alcon)	*2* *3* *3.75* *4.5*	2 mm 3 mm 3.75 mm 4.5 mm	0.45 0.50 0.57 0.62	0.26 0.23 0.18 0.20	0.29 0.27 0.25 0.18	0.33 0.40 0.47 0.43	0.14 0.16 0.15 0.12	0.20 0.17 0.15 0.12	21.00 20.95 20.82 20.88	2.48 2.15 2.13 2.27	3.39 3.25 3.34 3.31
TECNIS Synergy Optiblue (Johnson & Johnson VISION)	*2* *3* *3.75* *4.5*	2 mm 3 mm 3.75 mm 4.5 mm	0.35 0.58 0.34 0.40	0.26 0.00 0.29 0.19	0.39 0.42 0.38 0.40	0.27 0.31 0.19 0.21	0.20 – 0.16 0.10	0.30 0.22 0.21 0.21	20.96 20.73 20.69 20.73	2.64 – 2.51 2.70	3.32 3.44 3.53 3.56

Specifically, with the FineVision HP IOL, an MTF increase was observed compared to on-axis measures when the IOL was tilted 5º at 2 and 3 mm apertures (22.5 and 16%, respectively). Overall, the far MTF when tilting this IOL was almost equal at all apertures (0.4 for 2 mm and 0.45 for all other apertures). However, the intermediate and near MTF peaks had a steady drop with increasing apertures (Fig. 1, Table 3). In contrast, when the Acriva Trinova IOL was tilted 5º, far MTF dropped for 2, 3.75, and 4.5 mm apertures (7, 17, and 26%, respectively), while a relative maintenance of the MTF for the rest of foci was present. With the AT LISA IOL, 5° of tilt led to an MTF drop for apertures 2, 3, 3.75 and 4.5 mm (about 32, 32, 8, and 4%, respectively). In this case, resolution target images were very useful to see the level of blurriness induced with IOL tilt in the intermediate focus. Changes with IOL tilt with the PanOptix IOL were slight, except for the aperture of 3.75 mm for which the far MTF was 0.47, a higher value than that obtained with on-axis measurement (0.37) (Table 3). Resolution target images showed low clarity, especially for intermediate images at 3.75 and 4.5 mm apertures (Fig. 2). Finally, with the Tecnis Synergy IOL, resolution target images were very blurry in general after inducing the IOL tilt, with lower levels of MTF associated (Fig. 2, Table 3), but it must be noted here that the Tecnis Synergy IOL is the only tested that is designed the most aberration correction. Further studies would be needed to investigate the somewhat surprising results of tilted lenses.

### Optical performance data with IOL decentration

Regarding the impact of decentration, it was variable, even leading to some improvements in near MTF for the smallest aperture with the Acriva Trinova, PanOptix and Tecnis Synergy IOLs (Fig. 1). Specifically, when the Acriva Trinova IOL was decentered 0.5 mm, far MTF increased for the 2 mm aperture (about 3%), but it dropped for the apertures of 3 mm, 3.75 mm, and 4.5 mm (about 5%, 9%, and 16%, respectively) (Table 3). The 0.5 mm decentration of the FineVision HP IOL induced an increase of far MTF for the aperture of 2 mm (about 3%), whereas it dropped for 3 mm, 3.75 mm, and 4.5 mm apertures (about 5, 9, and 16%, respectively) (Table 4). With the AT LISA IOL, the decentration of the IOL mainly affected the intermediate optical quality, especially for larger pupils. In contrast, with the PanOptix IOL, the decentration mainly affected the far MTF, with a drop of this parameter for larger apertures (Fig. 1, Table 4). Regarding the Tecnis Synergy IOL, its decentration of 0.5 mm especially affected the intermediate focus obtained, with light levels of 0% for the apertures of 2 mm and 3 mm (Table 4).

**Table 4:** Optical performance data obtained in the experimental measures on-axis simulating a decentration of 0.5 mm with the Lambda PMTF device for the five intraocular lenses (IOLs) evaluated.

Trade Name (Company)	Aperture (mm)	Energy distribution			MTF			Lens power (D)		
		*Far*	*Int*	*Near*	*Far*	*Int*	*Near*	*Power*	*Int Add*	*Near Add*
Acriva Trinova Pro C (VSY Biotechnology)	*2* *3* *3.75* *4.5*	0.43 0.42 0.39 0.38	0.22 0.25 0.31 0.34	0.35 0.34 0.29 0.29	0.43 0.39 0.33 0.26	0.11 0.19 0.23 0.21	0.29 0.26 0.23 0.18	20.40 20.30 20.37 20.46	1.76 1.71 1.65 1.68	3.43 3.64 3.62 3.52
FineVision HP (PhysIOL)	*2* *3* *3.75* *4.5*	0.38 0.47 0.53 0.56	0.25 0.20 0.17 0.17	0.36 0.33 0.31 0.27	0.32 0.37 0.42 0.43	0.20 0.15 0.12 0.09	0.28 0.26 0.23 0.21	21.64 21.50 21.39 21.34	1.39 1.61 1.75 1.73	3.19 3.43 3.47 3.55
AT.LISA tri 839 M (Carl Zeiss Meditec)	*2* *3* *3.75* *4.5*	0.41 0.20 0.46 0.44	0.27 0.47 0.26 0.27	0.32 0.33 0.28 0.30	0.43 0.40 0.29 0.27	0.31 0.14 0.15 0.10	0.26 0.25 0.19 0.17	21.00 20.81 20.67 20.52	1.02 1.99 1.93 1.87	3.39 3.24 3.30 2.65
AcrySof PanOptix TFNT00 (Alcon)	*2* *3* *3.75* *4.5*	0.56 0.46 0.46 0.48	– 0.23 0.25 0.31	0.44 0.30 0.29 0.21	0.30 0.31 0.30 0.34	– 0.13 0.12 0.15	0.24 0.19 0.16 0.11	20.96 20.91 21.09 19.88	– 2.76 1.88 1.25	3.16 3.11 2.86 4.04
TECNIS Synergy Optiblue (Johnson & Johnson VISION)	*2* *3* *3.75* *4.5*	0.50 0.56 0.37 0.45	0.00 0.00 0.24 0.24	0.50 0.44 0.39 0.31	0.38 0.34 0.23 0.23	– – 0.15 0.12	0.38 0.27 0.24 0.16	21.21 20.94 20.74 20.60	– – 3.06 2.86	2.54 3.28 3.92 3.85

### IOL dimensions and diffractive disks analysis

**Figure 3:**
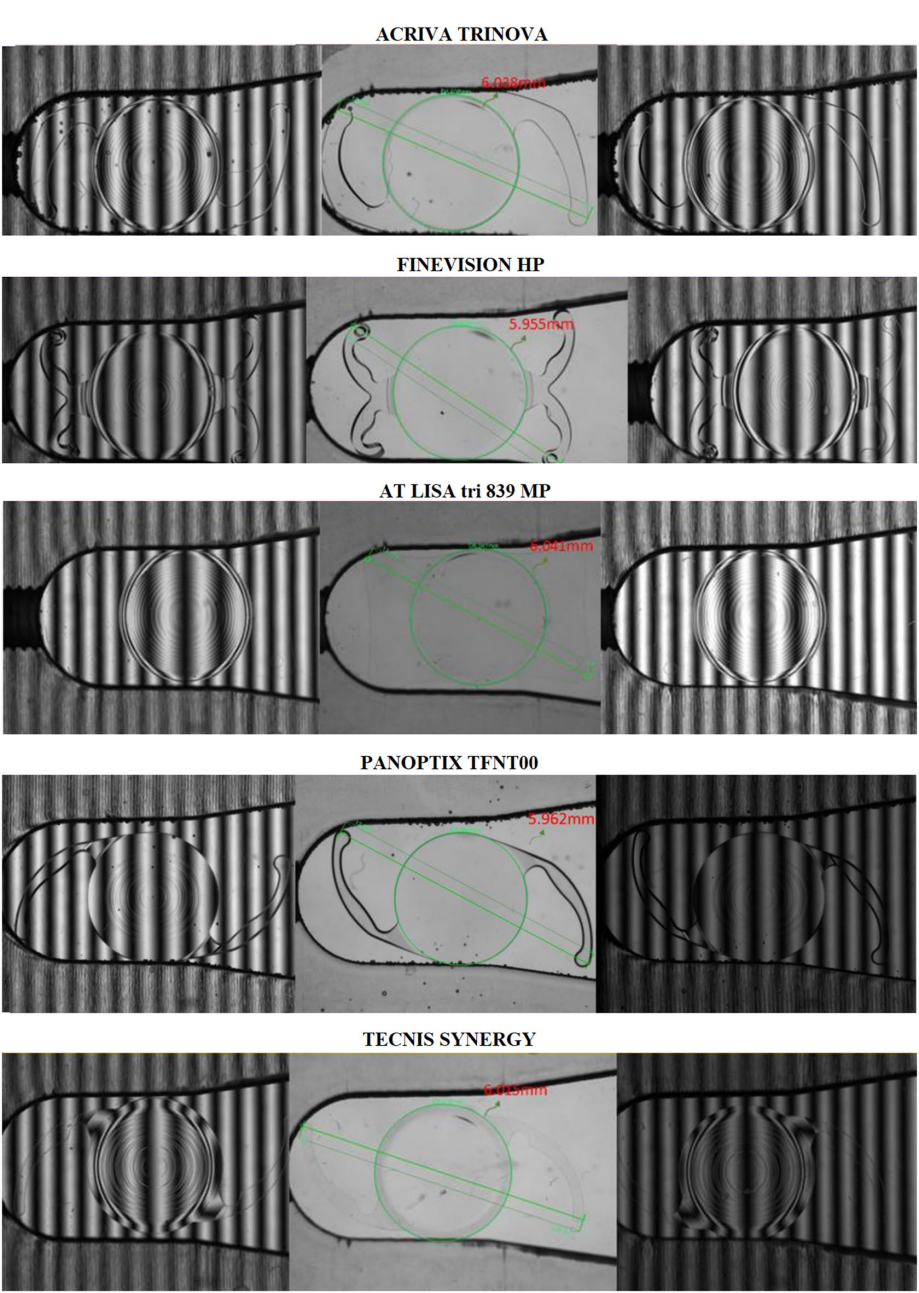
Analysis of the dimensions and diffractive disk profile of the 5 IOLs evaluated in the current experimental study using the VisIOLA system (Rotlex, Omer, Israel).

Figure 3 shows the anterior and posterior images obtained of each IOL using the VisIOLA system to analyse their dimensions and diffractive disk profile. All IOLs, as stated by the manufacturer, had an optic of approximately 6.0 mm of diameter. Specifically, the optic diameters measured were 6.038 mm, 5.955 mm, 6.041 mm, 5.962 mm, and 6.015 mm for the Acriva Trinova, FineVision HP, AT LISA tri, PanOptix and Synergy IOLs, respectively. The images obtained for the FineVision HP IOL showed that the diffractive disks got denser through the edge of the lens, with over 20 around the lens. Concerning the AT LISA IOL, one prominent feature of its optic design was its smooth diffractive circle design, compared to the sawtooth design of the rest of trifocal IOLs, except the Acriva Trinova IOL.

## Discussion

In the current experimental study, five different commercially available trifocal IOLs were evaluated in terms of optical profile and performance: Acriva Trinova, FineVision HP, AT LISA tri 839MP, PanOptix TFNT00, and Tecnis Synergy IOLs. In this study, a comprehensive benchmark analysis was conducted on the performance of several widely used multifocal lenses under tilted and decentrated conditions. The IOLs were measured under on-axis, tilted and decentrated conditions and MTF, USAF and energy distribution measurements were used to investigate the optical behaviour. Labuz et. al. measured and compared five trifocal IOLs, but only analysing their on-axis optical behaviours.^30^ Likewise, some studies have investigated the impact of IOL asphericity in the presence of IOL tilt and decentration^16, 31–34^. Pérez-Gracia et. al. tested 4 monofocal lenses and showed that aspheric lenses were more sensitive to tilt and decentration.^32^.

As previously mentioned, the Acriva Trinova IOL uses a sinusoidal diffractive surface design to distribute the light energy. The IOL topographic results in one previous study showed Fresnel disks with a mean height of 0.78 ± 0.10 μm, which is very close to the theoretical values.^18^ In the current study, the portion of light energy reaching the far focus remained almost constant at different apertures with this IOL. The MTF showed a dropping trend at near and far foci with increasing apertures. Compared to the other four IOLs, the Acriva Trinova IOL showed the largest intermediate MTF value for larger apertures. It should be considered that the intermediate vision in a sinusoidal design corresponds to 0^th^ diffractive order term in contrast to saw-tooth optic designs, which in most cases utilizes the 0^th^ order for the far vision.^18^ The FineVision HP features a saw-tooth design with the combination of two diffractive surfaces, and also combined with apodization that increases MTF at far focus.^19^ Indeed, in the current study, the performance of this IOL at the far focus was quite good, getting better at larger pupils (far MTF increased by 38% for increasing the aperture from 2 to 4.5 mm). However, the MTF at the intermediate focus was poorer compared to far and near at all apertures (MTF values of 0.26 or below). Similar optical performance results have been reported for the FineVision IOL by previous authors using different experimental setups (Table 5)^7, 8^.

**Table 5:** Optical performance data previously reported by other authors with the same trifocal IOLs compared in the current experimental study.

Aperture	Authors	Measuring system	Acriva Trinova			FineVision HP			AT LISA tri 839 MP			PanOptix TFNT00		
			*Far MTF*	*Int MTF*	*Near MTF*	*Far MTF*	*Int MTF*	*Near MTF*	*Far MTF*	*Int MTF*	*Near MTF*	*Far MTF*	*Int MTF*	*Near MTF*
3 mm	Khoramnia et al.^7^ Carson et al.^8^ Lee et al.^9^ Tandogan et al.^11^ Our study (2022)	OptiSpheric IOL Pro Custom Setup Custom Setup Custom Setup Lambda PMTF	– – – – 0.37	– – – – 0.17	– – – – 0.26	0.37 0.33 – – 0.39	0.16 0.14 – – 0.15	0.23 0.23 – – 0.26	0.39 0.34 – 0.39 0.33	0.15 0.13 – – 0.18	0.15 0.21 – 0.19 0.19	0.40 – 0.40 – 0.38	0.15 – 0.15 – 0.16	0.17 – 0.18 – 0.20
4.5 mm	Khoramnia et al.^7^ Tandogan et al.^11^ Our study (2022)	OptiSpheric IOL Pro Custom Setup Lambda PMTF	– – 0.29	– – 0.27	– – 0.22	0.51 – 0.50	0.09 – 0.10	0.22 – 0.18	0.31 0.26 0.24	0.13 – 0.16	0.21 0.18 0.16	0.42 – 0.42	0.14 – 0.14	0.17 – 0.16

No previous study evaluating the optical performance data with the Tecnis Synergy IOL. All measurements were performed for the spatial frequency of 50 Lp/mm.

The AT LISA tri IOL combines trifocal and bifocal diffractive areas in the optic for efficient energy distribution at large pupil diameters. According to the MTF curves, better optical quality was present for far, intermediate and near foci at the smallest aperture. This result is consistent with those found by Khoramnia et al^7^ and Tandongan et al^11^ in previous experimental studies (Table 5). Concerning the PanOptix IOL, it behaved as a bifocal lens for a 2 mm aperture, with increasing MTF values for the far focus and decreasing near MTF as the pupil aperture was larger. The intermediate optical quality remained almost constant from 3 to 4.5 mm apertures. This behaviour might be attributed to the elongated transition optic design and was also reported in other optical bench studies by Khoramnia et al^7^ and Lee et al. (Table 5)^9^.

The Tecnis Synergy IOL was introduced as a “Continuous-Range-of-Vision IOL”, being theoretically a multifocal IOL with enhanced depth of field property. Indeed, in clinical studies, this IOL was found to cover a larger vision range with spectacle independence.^23^ In the current experimental study, intermediate and near were found to join in one elongated peak for the smallest aperture. However, the MTF peaks corresponding to near and intermediate vision became clearly differentiated as the pupil aperture increased. It should be remarked that the optical quality of the intermediate focus was limited, especially for the largest aperture analysed, 4.5 mm. It should be also mentioned that the dioptric power also changed with this IOL as the aperture increased, experiencing a variation from 19.97 D to 20.75 D for 2 mm and 4.5 mm apertures. This can partly be attributed to spherical aberration in the lens. However, a value of -0.27 µm of spherical aberration for a 6 mm aperture would have been associated to an expected power shift between 2 mm and 4.5 mm of around -0.25 D and not -0.78 D. Then, the measured shift was larger than expected. No previous optical bench data of this IOL have been reported in the peer-reviewed literature and our results cannot be compared with previous experiences.

The clinical effect of IOL tilt and decentration is also crucial for understanding the behaviour of any multifocal IOL which is evaluated in optical bench. For this reason, this type of analysis has been also included. A comprehensive review study claims that in average, a tilt within ± 5° is very common in clinical practice when evaluating the postoperative position of an IOL and even is also common in the natural crystalline lens.^13^ For this reason, a 5º level of tilt was induced with each type of IOL and the optical effect was analysed, obtaining important differences between trifocal IOLs. In general, the most affected focus was that corresponding to far vision, with a reduction of its optical quality with increasing apertures. Furthermore, the intermediate focus was especially affected with IOL tilt for the FineVision HP, AT LISA, and PanOptix IOLs at larger apertures. With the Tecnis Synergy IOL, the limited optical quality for the intermediate focus was maintained, whereas the far and near MTF decreased, especially at larger apertures. Therefore, the impact of this potentially common level of IOL tilt can be significant in terms of optical performance of the IOL within the eye. Ruiz-Alcocer et al^5^ found negligible changes with 4º of tilt of the FineVision IOL using the PMTF device but simulating corneas with previous myopic laser refractive surgery.

Regarding the impact of 0.5 mm IOL decentration, it was quite limited in most of cases, to the extent that even improvements were recorded for far (Acriva Trinova, and FineVision HP) and near MTF (Acriva Trinova, PanOptix and Tecnis Synergy IOLs) with some IOLs at smaller apertures. Tandogan et al^11^ investigated the impact of IOL decentration with three IOLs, being one of them the trifocal AT LISA IOL. As in our experimental study, they found a degradation of the optical quality for intermediate focus. Specifically, intermediate MTF changed from 0.15/0.10 (3.0 mm/4.5 mm) to 0.12/0.08 after decentring the IOL 1 mm.^11^ In our study, intermediate MTF changed from 0.18/0.16 to 0.14/0.10 after 0.5 mm of decentration. The decentration of 0.5 mm of the Tecnis Synergy IOL also showed in our experience a significant degradation of the intermediate focus. In contrast, the decentration mainly affected the far MTF with the PanOptix IOL. It should be considered that those IOLs with the additional feature of the compensation for the corneal spherical aberration by inducing some level of negative spherical aberration may be especially limited by IOL decentration and tilting. Indeed, it has been demonstrated that the optical performance of aberration correcting IOLs can be markedly downgraded by misalignment and tilt.^31^ Therefore, this aspect may be one of the critical factors for the MTF degradation with some of the IOL models evaluated.

Finally, an analysis of the IOL design and dimensions was performed with the VisIOLA system, confirming that the optic size and the diffractive disk profile were consistent with the data provided by the manufacturer. Specifically, all IOLs had an optic of approximately 6.0 mm of diameter and showed a diffractive profile consistent with that described in the lens brochure of each IOL. Other previous studies have performed a more careful analysis of diffractive IOLs by using optical profilometry.^4, 19, 24, 35^ Loicq et al^19^ used an optical profilometer to characterize the surface topography of several IOLs, including the FineVision POD F, the PanOptix TFNT00 and the AT LISA tri 839 MP IOLs. These authors confirmed the all the trifocal diffractive IOLs evaluated presented a double diffractive structure, corresponding to a combination of two diffractive patterns, with the position of diffractive steps along the IOL radius depending on the square root of the step number.^19^.

This study has some limitations that should be acknowledged. First, measurements were carried out with monochromatic light, not white light that possibly is a condition closer to reality. However, we have followed strictly the ISO standards for characterizing the behavior of an IOL. Second, the number os IOLs tested may be considered as a limitation, but it is one of the optical bench studies for characterizing presbyopia-correcting IOLs with the highest number of IOLs compared that have been reported in last years. Possibly, in future studies, a comparison between trifocal and extended depth of focus IOLs can be performed. Furthermore, only one IOL sample of each type was measured. The addition of another IOL sample would have been useful to confirm the findings obtained, especially those that were unexpected. This should be confirmed in future experimental studies evaluating and comparing the optical behavior of multifocal IOLs. Finally, this is a laboratory study in which experimental data have not been correlated with clinical data. This could be an interesting future line of research, the link between optical bench and clinical visual performance data.

In conclusion, there are significant differences in the preclinical optical bench performance of the five trifocal IOLs evaluated and therefore clinical comparative studies must be performed to characterize the real clinical impact of such differences. In optical bench that utilizes aberration-less cornea model, all trifocal IOLs evaluated showed a far MTF performance over 0.3 at 3 mm aperture, except for the Tecnis Synergy IOL. FineVision HP and PanOptix lenses had better far MTFs for larger apertures while the Acriva Trinova Pro IOL had good intermediate and near vision for the same apertures, suggesting the potential benefit of mesopic visual performance at such distances with these IOLs. The induction of 5° of tilt or 0.5 mm of decentration led to different optical behavior depending on the IOL, confirming that the impact of these phenomena was not expected to be similar with each of the trifocal IOLs evaluated. Overall, decentration and tilt affected the energy distribution and caused MTF oscillations for different apertures. It should be noted that measurements in this study were made with an aberration-free cornea, which would have affected different lens designs differently. For a complete analysis of the optical behaviour of each IOL, more conditions need to be studied. There is much room for further studies to understand multifocal IOL performance under different conditions and for different eye optics.

## Data availability 

Data is available upon reasonable request to David P Piñero, david.pinyero@ua.es.
